# Factors associated with the prevalence of depression and anxiety among parents of children with neurodevelopmental disorders in Saudi Arabia

**DOI:** 10.1186/s12889-023-17228-9

**Published:** 2023-11-23

**Authors:** Ali J. Alsaad, Mujtaba M. Al Khamees, Abdulelah N. Alkadi, Majd A. Alsaleh, Aeshah S. Alshairdah, Zahra’a A. Alessa

**Affiliations:** 1 Department of Clinical Neuroscience, College of Medicine King Faisal University, Al-Ahsa, Saudi Arabia; 2 College of Medicine King Faisal University, Al-Ahsa, Saudi Arabia

**Keywords:** Neurodevelopmental disorders, Depression, Anxiety

## Abstract

**Background:**

Neurodevelopmental disorders (NDDs) are a group of conditions that include attention-deficit/hyperactivity disorders, specific learning disorders, autism spectrum disorder, intellectual disability, and other disorders. Raising a child with an NDD can be difficult because it affects the social lives of the parents and their relationships. It also requires the parents to develop another set of skills to deal with their child. These factors increase their risk of depression and anxiety.

**Aim:**

To measure the prevalence rates of depression and anxiety among parents of children with different NDDs, compare the rates between mothers and fathers, and measure the relevant associated factors.

**Methodology:**

This study was a prospective, qualitative, cross-sectional, anonymous questionnaire-based study. The participants were 416 parents of children with NDDs in Saudi Arabia. The sample size was determined using the Richard Geiger equation with a 5% margin of error, a 95% confidence level, and a 50% response distribution. The screening was performed using a validated Arabic version of the Patient Health Questionnaire-9 (PHQ-9) and Generalized Anxiety Disorder-7 (GAD-7). These are short and understandable screening tools that assist in identifying and grading the severity of depression and anxiety symptoms. The participants were reached by distributing the questionnaire to parents who followed up with Saudi NDD-related associations, clinics, and psychiatric clinics from November 20 to May 8, 2022. The data were collected, reviewed, and then entered into SPSS 21.

**Results:**

In total, 416 parents of children with NDDs in Saudi Arabia participated in the study. We demonstrated that 85.1% of parents of children with NDDs had depression and that 85.8% had anxiety. Mothers and fathers had similar rates of depression and anxiety. No significant difference was found between the type of NDD and rates of depression and anxiety in parents.

**Conclusion:**

Children with NDDs affect their parents’ mental health in terms of increased rates of depression and anxiety. This increase is not correlated with a specific etiology. Healthcare professionals who care for children with NDDs should also assess parental mental health and seek an early diagnosis of mental illness to ensure that the appropriate interventions are provided for parents.

## Introduction

Neurodevelopmental disorders (NDDs) are a group of conditions that include attention-deficit/hyperactivity disorders, specific learning disorders, autism spectrum disorder, intellectual disability, and other disorders [1]. They are associated with deficits in different domains, including the social, cognitive, language, and motor domains [2]. These conditions are usually seen before the age of 18 years [3]. Raising a child who suffers from an NDD can be challenging for parents, as it might affect their relationships and social lives. Furthermore, it might require more attention and a different set of parenting skills compared with raising a typically developed child, in addition to the need to learn about health services and become an advocate for their child. All of these factors could increase the parents’’ risk of developing psychological disorders, such as anxiety and depression [4, 5]. Furthermore, parents may become distressed when the expectations and wishes of their children can’t be met [3]. While there is an emphasis on mothers in the literature, both parents are affected and experience high levels of stress [2]. Four studies conducted in Arabic-speaking countries have discovered that parents of children with autism spectrum disorder had higher stress levels, decreased well-being, and other psychological issues than controls [6–9]. Other studies have examined factors related to psychological disorders and the stress faced by parents of children with autism spectrum disorders [10–12]. They have found that several factors appeared to affect the parents, such as financial distress, social support, and the presence of another caregiver.

Regarding depression and anxiety in particular, a recent Chinese study found that caregivers of children with neurodevelopmental delays are more likely to experience symptoms of depression, anxiety, or stress. Moreover, the prevalence of depression and anxiety is highly significant when the primary caregiver is older or has a low educational level [13]. Locally, an Omani study found that depression and anxiety rates are higher in parents of children with autism spectrum disorder than those of parents of children with other intellectual disabilities or parents of typically developed children [14]. In Arar, Saudi Arabia, 63.1% of caregivers of children with autism exhibited a reduction in quality of life, resulting in emotional problems. In Riyadh, similar results indicated higher levels of depression and anxiety in parents with children diagnosed with autism [15]. Regardless of the autism spectrum disorder, data are lacking regarding depression and anxiety among parents of children with other NDDs from studies that target both parents, which is the gap that we sought to fill. Therefore, this study aimed to measure the prevalence rates of depression and anxiety among parents of children with different NDDs, compare the rates between mothers and fathers, and measure the relevant associated factors.

## Method

### Study design

This study employed a qualitative, cross-sectional, anonymous questionnaire-based study design.

### Participants

In total, 416 parents of children with NDDs in Saudi Arabia were enrolled in this study. The sample size was determined using the Richard Geiger equation with a 5% margin of error, 95% confidence level, and 50% response distribution.

The inclusion criteria were any parents with a child or children diagnosed with NDDs in Saudi Arabia. The exclusion criteria were parents of typically developed children, parents who refused to be involved in the study, and parents who did not complete the questionnaire.

### Data sources and measurement

In this research on NDDs in Saudi Arabia, a collaborative approach was adopted to source data from various organizations and professionals. This approach aimed to ensure a comprehensive and diverse representation of the NDD landscape in different regions of Saudi Arabia by reaching out to associations and organizations that address NDDs across the country, covering a broad geographical spectrum. These associations included the Autism and Developmental Disorders Association in Makkah, the Autism Society in Tabuk, the Shamah Autism Center, and the Middle East Daycare Center.

Moreover, to delve into the educational aspect of NDDs, we collaborated with specialized teachers who possessed expertise in working with children with developmental disorders. Understanding the mental health dimensions of NDDs was a key focus of this research. For this purpose, we engaged with psychiatrists who practiced in various psychiatric clinics across Saudi Arabia. They were contacted by the research consultant responsible for this study. Their insights were invaluable in comprehending the psychological and psychiatric aspects of NDDs in different geographical regions. The data were collected through various channels, including email, websites, and official social media accounts of the associations.

The questionnaire contained five sections for capturing the sociodemographic data for parents (relationship with parents, age, educational level, occupation, financial income, history of chronic illness, and family history of any psychiatric diseases) and the affected child (child age, gender, diagnosed NDD, and age at diagnosis), followed by the PHQ-9 section and lastly the GAD-7 section. Parents were screened for depression and anxiety using the validated Arabic versions of the PHQ-9 and GAD-7. This study adapted and validated the PHQ-9, a widely used instrument for assessing depression symptoms, to ensure its suitability for the Saudi population.

The PHQ-9 and GAD-7 questions were statements that concerned how often certain things had been noticed over the last two weeks. The PHQ-9 statements included the following: One has little interest or pleasure in doing things; feels down, depressed, or hopeless; has trouble falling or staying asleep; sleeps too much; feels tired or has little energy; has a poor appetite or overeats; feels bad about oneself (or that one is a failure and has let oneself and one’s family down; has trouble concentrating on things (e.g., reading the newspaper or watching television); moves or speaks so slowly that other people could have noticed – or the opposite: being so fidgety or restless that one has been moving much more than usual; has thoughts that one would be better off dead; or hurts oneself [7]. The GAD-7 statements included the following: One feels nervous, anxious, or on edge; is unable to stop or control one’s worrying; worries too much about different things; has trouble relaxing; is so restless that it is difficult to sit still; becomes easily annoyed or irritable; and feels afraid, as though something awful might happen.

At the end of both questionnaires, there was a question regarding the extent to which these symptoms made it difficult for the person to do their work, take care of things at home, or get along with other people. The answer options were “not difficult at all,” “somehow difficult,” “very difficult,” or “extremely difficult.”

For the PHQ-9, the overall score was obtained by summing all discrete scores for the items, which ranged from 0 to 27 points. On the PHQ-9 scale, those who had scores of 0–4 were considered normal, 5–9 was mild depression, 10–14 was moderate depression, 15–19 was moderately severe depression, and 20–27 was severe depression. For the GAD-7, each item asked the individual respondent to rate the severity of his or her symptoms over the past two weeks. The response options were as follows: “not at all,” “several days,” “more than half the days,” and “nearly every day.” Scores of 5, 10, and 15 were set as the cut-off points for mild, moderate, and severe anxiety, respectively. Patients who scored 10 or more were considered positive for depression and anxiety.

Descriptive analysis was conducted by prescribing the frequency distribution and percentage for the study variables, including parents’ biodemographic data, children with NDD data, type of NDD, PHQ-9 items, and GAD-7 items, while anxiety and depression prevalence and severity were graphed. Cross-tabulation for depicting the distribution of parents’ depression and anxiety by their biodemographic data was conducted with the Pearson chi-square test for significance and the exact probability test for small frequency distributions.

### Ethical considerations

Ethics approval was obtained from the Research Ethics Committee at King Faisal University in Al-Ahsa, Saudi Arabia (KFU-REC-2021- NOV-EA000225). All methods were conducted in accordance with relevant guidelines and regulations. The participants responded anonymously to the online survey, and the e-questionnaires began with the respondents filling out an informed consent letter in the first section. This consent form informed participants about the confidentiality of their information. They were also provided with information concerning the research purpose and informed that they had the right to revoke their participation without prior justification.

### Data analysis

The data were collected, reviewed, and then entered into SPSS ver. 21 (IBM). All of the statistical methods used were two-tailed with an alpha level of 0.05; P values less than or equal to 0.05 were considered significant.

The Arabic version of the PHQ-9 was used, which exhibited strong internal consistency with a Cronbach’s alpha coefficient of 0.857. This confirmed its accuracy in measuring the intended aspects and established its validity for evaluating depression in Saudi parents. The GAD-7’s internal consistency was high, as measured by a Cronbach’s alpha coefficient of 0.763, which indicated strong reliability. Thus, the instrument was deemed valid for the Saudi population [16].

## Results

A total of 416 parents of children with NDDs completed the study questionnaire. Table 1 shows that the majority of the respondents were mothers. Parents’ ages ranged from 20 to more than 60 years, with a mean age of 29.2 ± 13.9 years. Most of the parents were university graduates. As for work, more than half of the parents were not working. A monthly income of more than 10,000 Saudi riyals (SR) was reported among 156 parents (37.5%), which was the highest percentage. Most parents had one to three children, of whom one child had NDDs. Only a third of the parents had a chronic health problem.

**Table 1:** Parent sociodemographic data of children with neurodevelopmental disorders (NDDs) in Saudi Arabia

Parents’ data	No	%
**Respondent**		
Father	119	28.6%
Mother	297	71.4%
**Parent’s age in years**		
20–40	267	64.2%
40–60	136	32.7%
> 60	13	3.1%
**Educational level**		
Below secondary	27	6.5%
Secondary	113	27.2%
University / above	276	66.3%
**Work**		
Not working	222	53.4%
Governmental work	122	29.3%
Private work	64	15.4%
Free work	8	1.9%
**Monthly income (Saudi riyals)**		
< 5000(less than 1335.82 US$)	120	28.8%
5000–10,000(1335.82–2671.74 US$)	140	33.7%
> 10,000(more than 2671.74 US$)	156	37.5%
**Number of children**		
1–3	269	64.7%
4–7	137	32.9%
> 7	10	2.4%
**Number of children with NDDs**		
1 child	370	88.9%
> 1 child	46	11.1%
**Chronic diseases**		
Yes	83	20.0%
No	333	80.0%

No.=number

Most of the parent respondents were mothers. The age range of parents was between 20 and more than 60 years old, with nearly two-thirds of the selected parents being aged 20–40 years. Nearly two-thirds were university graduates. Most were not working. Their monthly income varied between less than 5000 SR (less than 1335.82 US$) and more than 10,000 SR (more than 2671.74 US$), with almost a third of them having a monthly income above 10,000 SR. Nearly two-thirds of these parents had one to three children. Most parents had one child with an NDD. Most of them did not have any chronic diseases

Table 2 presents the sociodemographic data of children with NDDs. Almost half of the children (44.5%) were aged 6–10 years, while only 30 (7.2%) were aged 16–18 years. Most were boys. The most commonly diagnosed NDD was attention-deficit/hyperactivity disorder (ADHD), followed by autism, while the least diagnosed was intellectual disability. Almost half of the children (43.1%) were diagnosed by psychiatrists, and the diagnosis had been in the first 5 years of life among 291 children (70.1%), which was the highest percentage.

**Table 2:** Sociodemographic data of children with neurodevelopmental disorders (NDDs) in Saudi Arabia

Child’s data	No	%
**Child’s age**		
1–5	108	26.0%
6–10	185	44.5%
11–15	93	22.4%
16–18	30	7.2%
**Child’s gender**		
Male	319	76.7%
Female	97	23.3%
**Type of NDD**		
ADHD	254	61.1%
Autism	164	39.4%
Communication disorder	137	32.9%
Learning difficulty	83	20.0%
Motor disorders	65	15.6%
Intellectual disorder	53	12.7%
**How the child was diagnosed with (NDDs)**		
Psychiatrists	179	43.1%
neurodevelopmental specialized clinics	158	38.1%
Pediatrician	45	10.8%
Others	33	8.0%
**Age at child diagnosis**		
1–5	291	70.1%
6–10	99	23.9%
11–15	16	3.9%
16–18	9	2.2%

ADHD = Attention-deficit/hyperactivity disorder no.=number

There was Variation existed in the children’s ages, which ranged from 1 up to 18 years, with nearly half of them being aged between 6 and 10 years old. Most of the children were boys. The most commonly diagnosed NDD was ADHD, followed by autism and then, communication disorder. Most of them were diagnosed by psychiatrists or at neurodevelopmental specialized clinics; 10.8% were diagnosed by pediatricians. The bulk of children were diagnosed at the age of 1–5 years

Table 3 displays the depression and anxiety among parents of children with NDDs. Most of the parents had depression, which was mild among 21.5%, moderate among 25.5%, and moderate to severe among 38%. Furthermore, anxiety was detected among most parents, which was mild among 26.7%, moderate among 27.4%, and severe among 31.7%. Thus, most parents were found to have both depression and anxiety, while only 8.9% were free of these conditions.

**Table 3:** Depression and anxiety among parents of children with neurodevelopmental disorders (NDDs) in Saudi Arabia

Mental disorders	No.	%
**Depression**		
No	62	14.9%
Yes	354	85.1%
**Depression severity**		
Normal	62	14.9%
Mild	90	21.6%
Moderate	106	25.5%
Moderately severe	86	20.7%
Severe	72	17.3%
**Anxiety**		
No	59	14.2%
Yes	357	85.8%
**Anxiety severity**		
Normal	59	14.2%
Mild	111	26.7%
Moderate	114	27.4%
Severe	132	31.7%

No.= number

Most of these parents had depression, which varied in severity, with the largest portion of them having moderate depression. Moreover, most of them had anxiety, which varied in severity, with the largest portion having severe anxiety

**Fig. 1:**
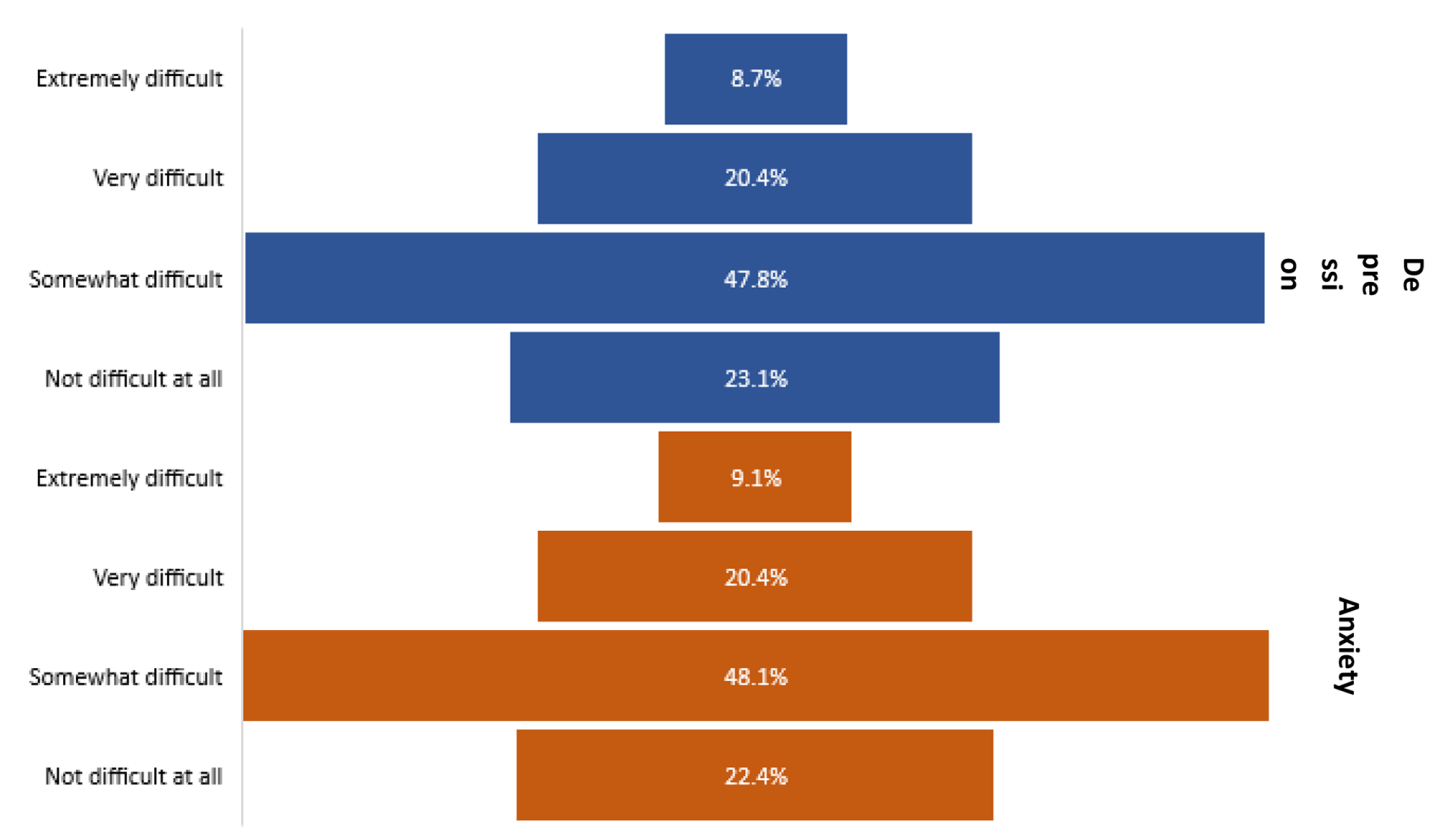
Results for the following survey question: How difficult have your experiences and problems made it for you to do your work, take care of things at home, or get along with other people? Note: The affection for depression and anxiety in parents’ lives was rated as having a severity level of “somewhat difficult.”

Figure 1 indicates the effects of GAD and/or depression on the parents’ lives in terms of work, study, household responsibilities, and relationships. Regarding depression, 29.1% of the parents experienced high to extreme difficulty in dealing with their activities or responsibilities. Regarding GAD, 29.5% of the parents experienced high to extreme difficulties in their daily lives. However, 23.1% and 22.4% of parents with depression and GAD, respectively, reported not experiencing any difficulties at all.

Table 4 presents the factors associated with parents’ depression and anxiety. Depression and anxiety had similar rates among mothers and fathers, with significant differences found to exist regarding depression as well as nonsignificant differences regarding anxiety. Depression was significantly higher among parents with more than one child with an NDD than those with one child with an NDD (97.8% vs. 83.5%, respectively; *P* = .010). Moreover, 92.8% of the parents with a chronic disease had depression, compared with 83.2% who did not have a chronic disease (*P* = .028). Additionally, 88.9% of parents of children diagnosed at the age of 6–10 years had depression, compared with 62.5% of parents of children diagnosed at the age of 11–15 years (*P* = .048). As for anxiety, it was detected among all parents with more than one child with an NDD compared with 84.1% of parents with one child with an NDD (*P* = .003). Furthermore, 92.8% of parents with chronic diseases experienced anxiety symptoms, compared with 84.1% of parents without such diseases (*P* = .024). Moreover, 92.9% of parents with a child diagnosed at the age of 6–10 years had anxiety, compared with 62.5% of parents with children diagnosed at the age of 11–15 years (*P* = .009).

**Table 4:** Factors associated with parents’ depression and anxiety

Factors	Depression		Anxiety	
	No	%	No	%
**Respondent**				
Father	106	89.1%	104	87.4%
Mother	248	83.5%	253	85.2%
*P-value*	0.149		0.559	
**Parent’s age in years**				
20–40	232	86.9%	231	86.5%
40–60	110	80.9%	113	83.1%
> 60	12	92.3%	13	100.0%
*P-value*	0.211		0.214	
**Educational level**				
Below secondary	20	74.1%	21	77.8%
Secondary	93	82.3%	97	85.8%
University / above	241	87.3%	239	86.6%
*P-value*	0.113		0.456	
**Work**				
Not working	182	82.0%	185	83.3%
Governmental work	109	89.3%	106	86.9%
Private work	55	85.9%	58	90.6%
Free work	8	100.0%	8	100.0%
*P-value*	0.182		0.287	
**Monthly income (Saudi riyals)**				
< 5000	104	86.7%	102	85.0%
5000–10,000	115	82.1%	117	83.6%
> 10,000	135	86.5%	138	88.5%
*P-value*	0.484		0.463	
**Number of children**				
1–3	228	84.8%	231	85.9%
4–7	118	86.1%	118	86.1%
> 7	8	80.0%	8	80.0%
*P-value*	0.842		0.865	
**Number of children with NDDs**				
1	309	83.5%	311	84.1%
> 1	45	97.8%	46	100.0%
*P-value*	0.010*		0.003*	
**Chronic diseases**				
Yes	77	92.8%	77	92.8%
No	277	83.2%	280	84.1%
*P-value*	0.028*		0.042*	
**Child’s gender**				
Male	276	86.5%	279	87.5%
Female	78	80.4%	78	80.4%
*P-value*	0.139		0.081	
**Child’s age at diagnosis**				
1–5	248	85.2%	247	84.9%
6–10	88	88.9%	92	92.9%
11–15	10	62.5%	10	62.5%
16–18	8	88.9%	8	88.9%
*P-value*	0.048*^$^		0.009*^$^	

*P* = Pearson X^2^ test $= exact probability test * *P* < .05 (significant) no.=number

Multiple factors were associated with parents’ depression and anxiety; one of them was gender, which had more significance in the development of depression. Another significant factor was the parent’s age. Educational level was significant in the development of depression but had the least significance in developing anxiety. Other significant factors were the work of the parents and the gender of the child. However, the number of children was of the least significance. Chronic diseases of the parents, having more than one child with NDD, and the child’s age at diagnosis have greater significance. Children diagnosed at 6–10 years contributed more to parents’ anxiety and depression than children diagnosed at age 11–15 years

Table 5 presents an analysis of depression and anxiety among subgroups of parents. The highest percentage of parents who had depression and anxiety was among parents of children with intellectual disorders, while the lowest percentage was among parents of children with motor disorders, and there were no statistically significant differences (*P* = .883).

**Table 5:** Analysis of depression and anxiety among subgroups of parents

Neurodevelopmental disorder	Psychological disorder								*p*-value
	Free		Depression		Anxiety		Both of them		
	No	%	No	%	No	%	No	%	
ADHD	22	8.7%	12	4.7%	13	5.1%	207	81.5%	0.883
Communication disorder	12	8.8%	8	5.8%	8	5.8%	109	79.6%	
Learning difficulty	6	7.2%	4	4.8%	5	6.0%	68	81.9%	
Intellectual disorder	4	7.5%	2	3.8%	3	5.7%	44	83.0%	
Autism	20	12.2%	11	6.7%	9	5.5%	124	75.6%	
Motor disorders	7	10.8%	3	4.6%	7	10.8%	48	73.8%	

*P* = exact probability test. ADHD = attention-deficit/ hyperactivity disorder. No.=number

Both depression and anxiety were more common in parents with children with intellectual disorders, and the percentage of depression and anxiety was slightly lower among parents with children with learning difficulties. It gradually started to reduce among parents of children with ADHD, communication disorder, autism, and motor disorders

Table 6 compares parents of children with one NDD versus parents of children with more than one NDD. Regarding anxiety and depression, most parents of children with two NDDs had depression and anxiety, compared with parents of children with only one NDD and parents of children with three NDDs or more, and there were no statistically significant differences (*P* = .594).

**Table 6:** Anxiety and depression among parents of children with one neurodevelopmental disorder (NDD) versus parents of children with more than one NDD

Number of NDDs	Psychological Disorder								*p*-value
	Free		Depression		Anxiety		Both of them		
	No	%	No	%	No	%	No	%	
One	19	8.4%	14	6.2%	16	7.1%	176	78.2%	0.594
Two	8	8.0%	3	3.0%	3	3.0%	86	86.0%	
Three / more	10	11.0%	5	5.5%	6	6.6%	70	76.9%	

*P* = Pearson X^2^ test NDD = neurodevelopmental disorder no.=number

Depression and anxiety were more common in parents of children with two NDDs than they were in parents of children with one NDD. Depression and anxiety were lower among parents of children with three or more NDDs, with no statistical significance (*P* = .594)

## Discussion

Parenting a typically developed child is a substantial challenge, and an even more significant challenge is parenting a child with an NDD. Therefore, the current study evaluated the psychological state of parents of children with NDDs. The results revealed a correlation of depression and anxiety with having a child with NDD. Undoubtedly, having a child with an NDD presents multiple difficulties that differ from those of having a typically developed child, including extra care, support, attention, level of dependence, and health system navigation. Raising a child with an NDD psychologically affects parents and causes emotional consumption. We found that raising one or more children with an NDD increases parents’ responsibilities, which increases their susceptibility to and severity of mental illness. This study’s findings confirmed this (*P* = .003).

Furthermore, we expected to find higher rates of depression and anxiety among mothers, as they are commonly more involved in caring for their children. However, this study demonstrated that fathers had similar rates of depression and anxiety to mothers. A possible explanation for this finding is that it could be due to advanced parenting methods that involve both parents in the care of children. In Saudi Arabia, this is a novel parenting style that has risen in popularity in recent years. The literature, both regionally and globally, is divided on this matter. In multiple studies, similar results have demonstrated that mothers and fathers of children with NDDs express anxiety, depression, and distress at similar rates [2, 5]. Researchers from Oman studied the mothers and fathers of children with ADHD and intellectual disabilities and demonstrated that both parents have similar rates of depression and anxiety. They speculated that this is likely due to the significance of familial and social connections in the community [14]. On the other hand, a study in Kuwait suggested that mothers of children with autism have higher rates of depression than fathers [6]. The authors believed that mothers perform most of the additional care and practical work that a child with an NDD requires.

Noteworthily, having a child with an NDD is not the only factor in being anxious or depressed. According to our study’s findings, most of the parents who suffered from depression had other daily challenges related to work, home responsibilities, relationships, or even co-morbidities. Coinciding with multiple international studies, this study shows that chronic diseases affect depression and anxiety rates in parents [4, 5]. We believe that a mixture of multiple factors increases the burden on the parent, one of which is the limitations on daily activities that accompany chronic diseases. Having a child with an NDD restricts parents’ lives. The combination of these two factors has been theorized to cause increased levels of depression and anxiety. Furthermore, many scholars agree with the stress model described by Dr. Reid, in which the stress that is inherent to having chronic diseases and parenting a child with an NDD accumulates, causing increasing levels of stress, which are – in most cases – unbearable and lead to depression and anxiety [17].

We expected that the rate of depressive disorder and/or anxiety in parents would vary according to the type of NDD of their child. However, our findings revealed that the rates of depressive disorder and/or anxiety were comparable among the different types of NDDs, with minimal variations that were statistically nonsignificant. One study revealed that parents of children with autism spectrum disorder or ADHD have a higher rate of parenting distress compared with parents of children with other NDDs [2]. In this study, we noticed fewer people diagnosed with NDDs at age 11–18 years, and the highest number of children with NDDs were diagnosed at age 1–10 years. This coincides with another study conducted in Saudi Arabia for autism [18] and ADHD [19]. We believe that this can be attributed to the lesser severity of NDD symptoms when they present in this age group, such that they go unnoticed by parents. Another explanation is that mental health services and specialized neurodevelopmental clinics have become more available around the country.

This study has some limitations. The first is the variation of the number of subjects in some categories. For example, a considerable gap existed between the number of children affected by ADHD (254) and those affected by learning difficulties (83); therefore, the findings might not be accurate for children with learning difficulties, since the number of subjects was less than that for the others. Second, the study’s findings regarding the intensity of the disorder’s symptoms and its relation to parental stress lacked depth. This level of intensity might have a substantial impact on the parenting stress associated with each NDD.

## Conclusion

This study aimed to measure the prevalence of depression and anxiety among parents of children with NDDs in Saudi Arabia. We found that parents of children with NDDs had significantly greater degrees of depression and anxiety. Moreover, we discovered that several factors, such as having children with intellectual disabilities or chronic illnesses, as well as having more than one child with an NDD, contribute to the severity of depression and anxiety. This has a noticeable effect on the difficulties that parents face daily, such as those related to work, study, relationships, and other responsibilities. Lastly, to ensure that appropriate interventions are included for parents, healthcare professionals who provide care for children with NDDs should also measure parental mental health and seek an early diagnosis of mental illness.

## Data Availability

The survey instrument used in this study to collect data on parental depression and anxiety in the context of neurodevelopmental disorders is available upon request from the corresponding author. Due to the sensitive nature of the data and the potential for participant identification, the raw survey responses and individual participant data will not be made publicly available.

## Abbreviations

NDDs: Neurodevelopmental disorders
